# Religious Struggle and Life Satisfaction Among Adult Christians: Self-esteem as a Mediator

**DOI:** 10.1007/s10943-020-01082-9

**Published:** 2020-09-10

**Authors:** Małgorzata Szcześniak, Celina Timoszyk-Tomczak

**Affiliations:** Institute of Psychology, University of Szczecin, Szczecin, Poland

**Keywords:** Religious struggle, Religious comfort, Life satisfaction, Self-esteem

## Abstract

The religious dimension of life represents an important source of human strength, meaning, and coping for many people. However, the religious life is not always “smooth and easy” and can be associated with weak personal adjustment, poorer psychological well-being, and lower satisfaction. Yet, besides the direct relationship between these variables, some researchers postulate the existence of an indirect association that has not been fully explained by various psychosocial mediators. The aim of the present study was to verify whether self-esteem could be a potential mediator between religious strain and life satisfaction. The sample consisted of 607 adult Christians (49.6% women) aged between 18 and 79. We used the Religious Comfort and Strain Scale, the Satisfaction with Life Scale, and the Rosenberg Self-Esteem Scale. Consistent with our hypotheses, life satisfaction positively correlated with religious comfort and was negatively associated with fear/guilt, negative emotions toward God, and negative social interactions surrounding religion. The same pattern of results was shown in the case of self-esteem. Moreover, the outcomes obtained from bootstrap sampling (5000) with a 95% confidence interval indicated a significant role of self-esteem as a mediator in all of the relationships between: (1) religious comfort and life satisfaction; (2) fear/guilt and life satisfaction; (3) negative emotions toward God and life satisfaction; and (4) negative social interactions surrounding religion and life satisfaction.

## Introduction

The religious dimension of life represents an important source of human strength, meaning, and coping for many people (Exline et al. 2014a, b; Hill and Pargament 2003). It may contribute to comfort and security (Wilt et al. 2016), be beneficial for mental (Rippentrop et al. 2005; You et al. 2019) and physical health (Rippentrop et al. 2005; Tiggemann and Hage 2019), increase life satisfaction (Villani et al. 2019), act as a protective factor against substance abuse (Isralowitz et al. 2018; Van der Meer et al. 2008), suicide (Gearing and Alonzo 2018; Lester 2017), and depression (Ronneberg et al. 2016). For various people, religion is an important sphere that may affect other domains such as interpersonal relationships and the workplace (Exline 2013).

However, the religious life is not always “smooth and easy” (Hill and Pargament 2003, p. 69). At various stages of life development, different individuals may also experience religious struggles manifested in negative thoughts, beliefs, emotions or behaviors toward God and other religious people or institutions (Exline et al. 2014a, b). Several recent findings suggest that supernatural, interpersonal, and intrapersonal religious strains may negatively influence other dimensions of life. For example, feelings of alienation from God, and religious fear are listed as some indicators of psychological distress (Abu-Raiya et al. 2016; Exline et al. 2000; Zarzycka et al. 2019). Struggles with religiosity may also impede progress in some physical rehabilitation inpatients (Fitchett et al. 1999). Negative attributions of God’s intent appear to be a predictor of lower well-being (Wilt et al. 2016). Most studies show that religious struggle has a tendency to be associated with weaker personal adjustment (Zarzycka and Zietek 2018), poorer psychological well-being (Stauner et al. 2019), and lower life satisfaction (Park et al. 2011; Szcześniak et al. 2019). Preserving negative feelings of guilt/fear toward God and the religious community has been found to correlate negatively with life satisfaction (Szcześniak et al. 2019). Likewise, anxious and avoidant forms of attachment to God have been adversely related to satisfaction with life among Christian young adults (Zahl and Gibson 2012). In brief, the research literature seems to indicate that struggles relate to lower functioning (Wilt et al. 2016).

Yet, besides the direct relationship between these variables, some researchers postulate the existence of an indirect association that has not been fully explained by various psychosocial mediators (Cummings and Pargament 2010; Pargament et al. 2001). For example, Koenig (2012) suggests that religion influences human functioning through many different mechanisms. Among the most important mechanisms, the author mentions: cognitions that provide a sense of control over events and meaning to challenging life conditions, rules and regulations that reinforce constructive, prosocial behaviors and weaken destructive ones, psychological traits and virtues. Although Jones (2004) argues that the presence of mediators would help explain more accurately the ways in which religion really does affect human life, relatively little attention has been given to the nature of the relationship between religious struggles and well-being or satisfaction with life (Abu-Raiya et al. 2018; Park et al. 2012; Zarzycka and Puchalska-Wasyl 2019; Zarzycka and Zietek 2018). So far, we know that social support (Chang et al. 1998; Park et al. 2012; Pearce et al. 2006), hope (Zarzycka et al. 2019), gratitude (Szcześniak et al. 2019), the tendency to forgive (Zarzycka 2019), altruism (Lancaster and Miller 2019), and gender (Zukerman et al. 2017) are among the different explanatory mechanisms that mediate or moderate the effect of religious struggle on quality of life. Therefore, in our study, we assumed that self-esteem could be a potential mediator between religious strain and life satisfaction, since it has been found to play a mediating role in the relationship between religious beliefs and mental health (Beshlideh et al. 2009), and negative religious coping and irregular behaviors (Kim 2006; Latzer et al. 2014).

### Religious Struggle

Religious struggle, sometimes referred to in the literature as religious strain, spiritual dryness, distress related to faith, or negative religious coping, is perceived to be a manifestation of a less secure relationship with God or a higher power (Büssing et al. 2013; Exline and Rose 2005; Hebert et al. 2009; Pargament et al. 2000). Most contemporary researchers consider religious struggle to be a multidimensional construct. In Pargament’s conceptualization, religious struggle is a type of negative coping that reflects spiritual tensions with the divine, others, and within oneself (Pargament et al. 2011). People who negatively deal with stressful events that are related to the sacred (Pargament and Abu-Raiya 2007) tend to report a less secure relationship with God and reinterpret the stressor as God’s punishment. By contrast, positive religious coping strategies apply to a more secure relationship with God, perceived as loving and caring (Pargament et al. 2011). In Exline’s approach, religious struggle is not a coping reaction, but an experience; a kind of human feature. Exline et al. (2014a) distinguish three main domains of religious strain. First, “supernatural struggle” implies negative emotions and beliefs about God or to evil spirits that are considered to harmfully affect human life. In “divine struggle,” divinity is perceived as cruel or distant (Exline et al. 2014a), and the relationship with the sacred as troubled or conflicting (Bradshaw et al. 2010). In “demonic struggle,” malevolent supernatural agents are felt as threatening. Second, interpersonal struggle encompasses negative experience with one’s own religious community members, other groups, or institutions on religious issues. People may experience this kind of strain because of a lack of an adequate example, unpleasant interactions, or a difference of opinions in religious settings (Ellison and Lee 2010). Third, intrapersonal struggle denotes one’s own chronic and unresolved thoughts or wrestles that refer to moral principles, doubts, and ultimate meaning. Moral conflicts can be related to non-compliance with the principles of faith and expressed through remorse, shame, and embarrassment (Sedlar et al. 2018). Doubts are connected to queries about one’s own beliefs and religious involvement. Ultimate meaning applies to the loss or lack of deep meaning in once’s own life or in the world.

Spiritual dryness is another form of spiritual struggle (Büssing et al. 2017a). According to Büssing and colleagues (2016, 2017b, 2018), spiritual dryness refers to a variety of perceptions and emotions related to one’s own faith and spiritual life. Thus, people who endure this specific religious crisis may feel distant from or abandoned by God, undergo through spiritual emptiness, and experience incapacity to get in contact with God through prayer. Although this concept seems to have a negative connotation, which can lead to alienation from God, in the theological literature, it can be considered to be a phase of purification and growth, as well (Büssing et al. 2018).

A growing body of research has revealed that many people face struggle around religious aspects at various stages of their lives (Exline et al. 2014b; Van Tongeren et al. 2019). For example, Carter (2019) observed that religious struggle is a normal aspect of early adulthood and may represent a possibility to develop beliefs related to personal growth. Johnson and Hayes (2003) found that almost 25% of 5472 university students reported substantial distress related to religious and spiritual concerns. Moreover, negative forms of religiousness, while less frequent than positive religious expressions, arise also among some older adults (Pargament et al. 2001). Furthermore, religious struggle, just like the other forms of religiosity, occurs around the world and across different religious groups. Empirical studies confirm the existence of religious problems and tensions among Christians (Bryant and Astin 2008; Büssing et al. 2013, 2016, 2017a, b, 2018; Carter 2019; Łowicki and Zajenkowski 2017; Szcześniak et al. 2019; Zarzycka 2018; Zarzycka et al. 2017), Muslims (Abu-Raiya et al. 2008, 2015, 2018; Ai et al. 2003), Jews (Abu-Raiya et al. 2016; Pirutinsky et al. 2011; Pirutinsky and Rosmarin 2018; Rosmarin et al. 2009, 2017), Hindus (Benson et al. 2011; Exline et al. 2017; Simha et al. 2013; Tarakeshwar et al. 2003), and atheists or agnostic samples (Bradley et al. 2016; Sedlar et al. 2018).

In the present study, religious struggle was operationalized as a concern with one’s own transgressions toward God, feelings of being unforgiven by Him, and negative social interactions surrounding religion (Exline 2013). Such religious strain may be characterized by tensions, distress, and conflicts about the sacred sphere within oneself, with the supernatural, and with others (Exline and Rose 2005). In the personal aspect, religious struggle refers to the sense of guilt or doubts arising from one’s own wrongdoings against divinity. In the divine dimension, religious struggle includes negative emotions toward God resulting from adverse or unjust events that He allows. In the interpersonal component, religious struggle involves negative feelings and reactions to improper or harmful behaviors of clergy or other members of religious institutions and communities (Zarzycka et al. 2020).

### Religious Struggle and Life Satisfaction

Over the last few decades, life satisfaction has been examined by a remarkable number of studies around the world (Lachmann et al. 2018) built on different approaches to this concept (McClelland 2010). For example, according to the Quality-of-Life Theory, life satisfaction is defined in terms of needs, goals, and wish fulfillment (Frisch 1998). Moreover, satisfaction within the Multiple Discrepancies Theory (Michalos 1985) implies appraisals about the discrepancy between what people have or want and various standards. Veenhoven (2012) connects life satisfaction with the flow of positive and negative experiences. In turn, Pavot and Diener (2008) understand life satisfaction as a cognitive component of subjective well-being, and this conceptualization is assumed in the present study.

The top-down perspective of life satisfaction suggests that a global judgment of subjective well-being depends on personality and other stable traits (Diener 1984). Religion seems to be one of those dimensions of human functioning which may contribute to quality of life to a large degree. Different studies show that strong believers are happier than those who declare lower levels of religiosity (Krok 2014; ten Kate et al. 2017; Yeniaras and Akarsu 2016). Nevertheless, religion has been portrayed not only as a source of comfort, but also of strain (Exline et al. 2000). There is a growing body of research indicating that negative feelings toward the divine or religious community are often linked with lower levels of life satisfaction (Abu-Raiya et al. 2018; Szcześniak et al. 2019). Zarzycka and Zietek (2018) found that divine, demonic, interpersonal, moral, doubt, and ultimate meaning domains of religious struggle correlated negatively with life satisfaction, confirming some previous studies conducted among various groups of patients (Abu et al. 2019; Manning-Walsh 2005; Park et al. 2011; Ursaru et al. 2014). Since life satisfaction is a broad concept (Loewe et al. 2014) and dissatisfaction with life manifests itself in some poor health outcomes (Gigantesco et al. 2019), it is valuable to investigate how religious struggle relates to some indicators of life satisfaction. A systematic review by Braam and Koenig (2019) shows that 59% of studies analyzed by the authors indicate a significant increase of depressive symptoms in the context of religious struggles. Moreover, religious struggle also seems to predict a greater risk of mortality (Pargament et al. 2001), emotional distress (Fitchett et al. 2004), congestive heart failure (Park et al. 2011), and poorer recovery of activities in daily life (Fitchett et al. 1999).

Some researchers (Hill and Pargament 2003; Stauner et al. 2016; Zarzycka and Puchalska-Wasyl 2019) point out that the process of religious disappointment with God, people, and institutions may be linked not only to negative, but to positive outcomes, as well. Indeed, religious struggle can be a key to spiritual growth and development (Büssing et al. 2013; Hill and Pargament 2003). According to most religious and spiritual traditions, a time of deep religious strain is frequently a prolog to transformation and strengthening (Magyar-Russel and Pargament 2006). However, not every experience of religious or spiritual struggle is associated with positive outcomes. Hill and Pargament (2003) observe that depending on how capable people are at solving their religious strain, they may find and take the road that leads to growth or to decline.

### Religious Struggle, Life Satisfaction, and Self-Esteem

There is a growing body of evidence suggesting that spiritual struggle affects psychosocial functioning (Wilt et al. 2016). However, research is required to reveal why and how tensions around one’s own religiosity or spirituality may affect life satisfaction. Since interactions between religious strain and different dimensions of well-being are somewhat complex and are frequently influenced by “third” factors (Pargament et al. 2005), we decided on a research design including self-esteem as an underlying psychological mechanism of this relationship. The choice was dictated by the fact that self-esteem, conceptualized as an individual overall evaluation and respect of the self (Rosenberg 1965), can act as a buffer against adverse experience, and decrease the influence of negative life events (Mann et al. 2004; Nartova-Bochaver et al. 2019). Indeed, Pearlin and Schooler (1978) considered self-esteem to be one of the features of personality that people use to help themselves endure dangers posed by events and conditions in their environment. In this sense, self-esteem functions as a coping resource and protective factor during life hardships (Moksnes and Espnes 2013). Baumeister et al. (2003) suggested that it would be plausible to think of high self-esteem as enabling people to recuperate quicker or entirely from misfortunes and setbacks.

The research on self-esteem shows that people differ in the dimensions on which they ground their self-worth (Park et al. 2006). Some of them derive their sense of self-esteem from their academic competency or appearance, others build their general judgment of themselves on love, support of family, or even of their relationship with God (Crocker and Park 2003). A review of the literature shows that the relationship between religiosity and self-esteem can be viewed through the prism of various psychological perspectives. For example, Buri and Mueller (1993) investigated the association between one’s concept of God and their self-esteem within psychoanalytic theory. More specifically, their results showed that a perception of God as loving was correlated with the respondents’ self-esteem. Reinert and Edwards (2014) studied the religiosity/self-esteems relationship, using attachment theory that combines different levels of self-esteem with various kinds of interpersonal bonds. Their outcomes indicated that while the dimension of a loving God was positively related to the self as loving, it was negatively connected to a controlling and distant self. Inversely, the dimensions of a controlling and distant God were positively associated with a controlling and distant self, and negatively linked to a loving self. In other studies, there was a relationship between one’s own idea of God and one’s own self-concept (Myers 2008). Thus, having the perception of a loving, accepting, and caring God enhanced self-esteem. Similarly, Benson and Spilka (1973) found that loving-accepting images of God were positively related to self-esteem among Catholic participants, while rejecting images were associated negatively. Likewise, data obtained from Scottish adolescents led to the conclusion that images of God as cruel and punishing were correlated with lower self-worth (Francis et al. 2010). Moreover, religious participation enhanced self-esteem among suddenly bereaved family members (Sherkat and Reed 1992). In a study by Park and colleagues (2018), conducted among African American community-dwelling adults, a negative religious coping style not only correlated negatively with self-esteem, but was also its predictor. Altogether, Wilt et al. (2016) found that negative attributions of God’s intent, expressed through a sense of being turned away from, betrayed, abandoned, neglected, and abused, together with less meaning, and greater perceived spiritual decline, predicted poorer life satisfaction and self-esteem.

Besides its association with religious comfort and struggle, self-esteem positively relates to subjective outcomes for life satisfaction, being its robust predictor mainly in Western culture (Diener and Diener 1995). Beliefs about oneself reflect the extent to which people evaluate themselves as being worthy and valuable against negative or difficult experiences (Nartova-Bochaver et al. 2019). Much of the research conducted to date on quality of life provides support for self-esteem as an important factor for retaining psychological well-being during adolescence and various stages of adulthood (Moksnes and Espnes 2013). Taking into account the literature mentioned so far, we assumed that religious struggle could likely be associated with life satisfaction by means of the sense of self-esteem since the latter is a buffer against stress (Baumeister et al. 2003) resulting from experience of negative attitudes toward God and others on religious issues (Park et al. 2011).

### Research Problem and Hypotheses

In this study, we aimed to verify whether the relationship between religious struggle and life satisfaction was mediated by self-esteem. While there is enough evidence that religious strain may worsen quality of life (Exline and Rose 2005), the means through which religious struggle can increase psychological adjustment and lead to constructive effects is still understudied and less confirmed (Zarzycka and Zietek 2018). Thus, based on the literature assessment, we expected that:

#### **Hypothesis 1 (H1)**

Religious comfort is positively associated with life satisfaction, and other dimensions of religious struggle (fear/guilt, negative emotions toward God, negative social interactions related to religion) are negatively correlated with life satisfaction.

A theory that can form the basis of hypothesis H1 is attachment theory. In this regard, Kirkpatrick and Shaver (1992) observe that people’s beliefs about God and a personal connection with Him may be comparable to human attachment bonds. When relations with attachment figures are consistently good enough, the internal and external world of a child become safe and reliable (Homan 2014). In contrast, when these relations are generally inconsistent, such worlds turn out to be uncertain and unreliable. Therefore, the perception of God as a confident, secure, and caring attachment figure may be positively associated with different aspects of psychological well-being (Bradshaw et al. 2010; Culver and Lundquist Denton 2017). The foundation of hypothesis H1 lies also in the empirical research literature. One the one hand, people who tend to describe their relationship with God as secure, also report greater life satisfaction and lower anxiety (Kirkpatrick and Shaver 1992). On the other hand, individuals who state being anxiously or avoidantly attached to God, are inclined to present lower satisfaction with their lives (Homan 2014). Moreover, more recent studies (Wilt et al. 2016; Szcześniak et al. 2019) clearly show a negative association between religious struggles and life satisfaction. In fact, Wong et al. (2018) notice that struggles in the realm of the sacred can be very problematic. When one’s connection to the divine is broken or unhealthy, individuals may face difficulty which leads to negative outcomes.

#### **Hypothesis 2 (H2)**

Religious comfort is positively related to self-esteem, and other dimensions of religious struggle (fear/guilt, negative emotions toward God, negative social interactions related to religion) correlate negatively with self-esteem.

The rationale behind hypothesis H2 can be traced back to the theory of attribution. According to several writers, attributions are made to preserve and/or increase self-esteem (Spilka et al. 1985). If God is thought to view people as unworthy sinners, it is plausible to presume a negative association between religion and self-esteem. Conversely, if God is perceived as gratuitously loving and unconditionally accepting, it is reasonable to suppose a positive correlation between religion and high self-esteem (Jones and Francis 1996). This line of reasoning leads to the hypothesis that a perception of God as benevolent, caring, and trustworthy is related positively to self-esteem, and a view of God as harsh and merciless, is related negatively to self-esteem. With respect to fear/guilt struggle, Krause (1995) observes that religious guilt shapes self-esteem. More precisely, religious discontent and punitive religious reappraisals correlate negatively with self-esteem (Park et al. 2018). This may be due to the fact that susceptibility to guilt is related to God’s image perceived as punitive and vindictive (Hood 1992). In fact, guilt over a committed transgression is negatively associated with emotions and beliefs about oneself (Grubbs et al. 2016). Therefore, people who fear God and feel guilty may display a lower overall sense of self-worth. In regard to interpersonal struggle, Grubbs et al. (2016) find that self-esteem negatively correlates with interpersonal struggles, involving conflict with other people in a spiritual context. Thus, it can be assumed that feeling misunderstood by religious people or being angry at institutional religion may inversely correlate with positive self-conception.

#### **Hypothesis 3 (H3)**

Self-esteem mediate the effect of religious comfort and three dimensions of religious struggle on life satisfaction.

While the “strain hypotheses” (H1 and H2) are already well acknowledged, the “buffer hypothesis” (H3) can generate some doubts, as the suitability of the cross-sectional non-experimental design presents some methodological shortcomings (David and Sava 2015). Nevertheless, Kline (2015), and Fairchild and McDaniel (2017) admit that an indirect effect can be established when there is considerable justification for temporal ordering conditions of the studied variables (antecedents vs. outcomes): religious struggle and life satisfaction; religious struggle and self-esteem; self-esteem and life satisfaction. Different authors present some convincing theoretical frameworks that delineate the multiple pathways through which various dimensions of religious struggle may relate to well-being. For example, Park (2007) mentions that people’s perceptions, meaning systems, responses, decisions, and behaviors are among potential mechanisms that can mediate the relationship between religious strain and psychological well-being. Moreover, Greenberg and colleagues (1986) suggest that self-esteem may provide protection against fear and threats. People who pursue self-esteem and gain self-worth are more likely to use their personal resources to manage difficult experiences also within the domain of religious strains. Therefore, self-esteem can have anxiety-buffering qualities (Maxfield et al. 2014) that help individuals who are undergoing religious struggles to deal with negative emotions toward themselves, God, and the religious community.

Besides the theoretical rationale, there is some empirical evidence that self-esteem may be one of the pathways through which spirituality contributes to well-being (Joshanloo and Daemi 2015). Firstly, recurrent religious strains felt toward God, people or institutions can impact personal well-being (Mahoney and Cano 2014). Secondly, although the literature pertinent to the assessment of the association between religion and self-esteem provides quite conflicting findings (Krause 1995), some outcomes suggest that greater religious commitment tends to enhance feelings of self-worth. For example, a positive attitude toward Christianity, a positive image of God, and church attendance predicted higher self-esteem scores among Welsh adolescents (Jones and Francis 1996). Conversely, the characteristics of religious struggles, such as attributions of God’s role, perceived meaning, and spiritual growth/decline were predictors of self-esteem even when controlling for personality traits and religiousness (Wilt et al. 2016). Zinnbauer and Pargament (1998) observed that college students who underwent the process of spiritual conversion replaced their negative ideas about themselves with a higher self-esteem. Thirdly, self-esteem has been constantly found to strongly predict life satisfaction, both in individualistic and collectivistic cultures (Chen et al. 2006).

## Method

### Participants and Procedure

The sample consisted of 607 adult Christians (49.6% women) aged between 18 and 79. The average age was around 28 (*M* = 28.16; SD = 11.41). In terms of subjective assessments of their own levels of adherence to religiosity, 10.5% of participants declared a lack of religious involvement, 13.2% %—low, 15%—medium, 29.8%—rather high, and 31.5%—very high.

The participants were enrolled exclusively through selected online Internet communities and groups for Christians, mainly through the Facebook service. The motive for selecting this particular research group was because there is relatively little research on Christians with respect to this issue in the countries of Central Europe, although they are the largest religious population in Poland (Łowicki and Zajenkowski 2017). All of the respondents who decided to take part in the study were provided with general communication about the research objectives and were given a web-based informed consent. Only after offering their voluntary agreement, the participants were encouraged to complete the questionnaires.

### Measurements

The Religious Comfort and Strain Scale (RCSS), designed by Exline and colleagues (Exline et al. 2000) and adapted into Polish by Zarzycka (2014), is a questionnaire of 28 items that measures religiosity as a source of comfort (one subscale) and strain (three subscales). Participants evaluate each item on an 11-point Likert scale (from 0 = not at all to 10 = extremely) (Zarzycka 2014). Religious comfort regards the sense of trust in God, and the feeling of God as almighty and compassionate. It measures perceiving faith as a source of power, sense and purpose in life (Harris et al. 2015; Zarzycka et al. 2017). The subscale of fear and guilt relates to concern with one’s own transgressions and feelings of being unforgiven by God (“Belief that sin has caused your problems”). The subscale of negative emotions toward God denotes a feeling of alienation from God and a sense of being punished or condemned by God (“Feeling that God is far away”). The subscale of negative social interactions surrounding religion evaluates the intensity of adverse emotions and relations with the family, clergy, religious community (“Bad memories of past experiences with religion or religious people”). In the present study, the α of religious comfort was 0.98, of fear/guilt was 0.81, of negative emotions toward God was 0.85, and of negative interactions surrounding religion was 0.79.

The Satisfaction with Life Scale (SWLS), originated by Diener et al. (1985) and adapted into Polish by Juczyński (2001), is a short 5-item scale that evaluates one’s life satisfaction globally (“So far I have gotten the important things I want in life”) rather than specific in nature (Pavot and Diener 1993). The respondents assess each of the five statements by using multiple-choice answers on a 7-point Likert scale that ranges from 1 = strongly disagree to 7 = strongly agree. The higher the total score, the stronger the general satisfaction with life. In the current sample, the mean SWLS score was *M* = 21.07 (SD = 6.95), which is fairly comparable to results reported by other Polish samples (*M* = 21.14, Piotrowski and Kubacka 2015; *M* = 22.83 and *M* = 20.78, Jankowski 2012). Various studies report a good coefficient alpha of 0.82 (Diener et al. 1985). In the present study, Cronbach’s alpha was 0.85, and a single factor emerged which accounted for 63% of the variance of the scale.

The Rosenberg Self-Esteem Scale (SES), developed by Rosenberg (1965) and adapted into Polish by Łaguna et al. (2007), known for its relative simplicity and accessibility (Schmitt and Allik 2005), is a ten-item self-report instrument for evaluating individual feelings of self-worth or self-acceptance (“I feel that I am a person of worth, at least on an equal plane with others”). Respondents endorse each item on a 4-point Likert-type scale, rating from 1 = strongly disagree to 4 = strongly agree. Five items are negatively worded and require reverse coding. Scores range from 10 to 40, where 40 indicates the highest level of self-esteem. In the current sample, the mean result obtained was *M* = 28.94 and was slightly lower than the average self-esteem found in 53 countries (*M* = 30.85, Schmitt and Allik 2005). Cronbach’s reliability coefficient across all 53 nations was *α* = 0.81 (Schmitt and Allik 2005), in the adapted version, the α was between 0.81 and 0.83 (Łaguna et al. 2007), and in the present study, the α was 0.88.

### Data Analysis

In this study, data analyses were carried out using the Statistical Package for the Social Sciences (SPSS software version 20, IBM) with significance accepted if *p* < 0.05. Missing data were avoided as responses to the questions were mandatory in order to move on to the next division of scales. The assumptions of a normal distribution were checked through the statistics of skewness and kurtosis (Morgan and Griego 1998). Descriptive statistics were calculated, and Pearson’s correlation coefficients (*r*) for parametric data were evaluated to investigate the association between variables including religious struggle, life satisfaction, and self-esteem.

Next, we conducted a linear regression model to monitor for different possible confounding variables and to confirm if they were not threatening the validity of the mediation analyses. First, since four dimensions of religious comfort and strain could be associated with each other and used as predictors while accounting for covariates, we scrutinized whether there would be a high redundancy among predictors, or a multicollinearity problem. For this purpose, we used index of tolerance statistics and variance inflation factors (VIF). Second, we checked the data for outliers, using Mahalanobis’ distance and Cook’s distance. Third, the participants’ sex, age, and subjective assessment of their religious involvement were added to control for their possible effect on the relationship between the independent variable of interest (religious struggle) and the outcome variable (life satisfaction). Indeed, both theoretical and empirical analyses of the influence of religious struggle in relation to demographic variables found differences between women and men, and younger and older participants (Cokley et al. 2013). The potential confounders were entered at Step 1. All variables assumed as predictors of life satisfaction were entered at Step 2.

**Fig. 1:**
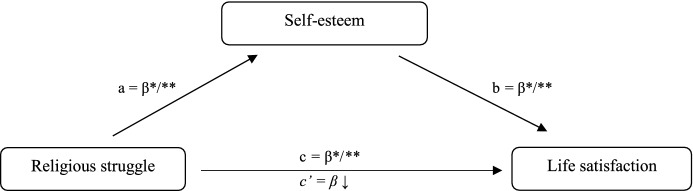
Theoretical model of the role of self-esteem in the relationship between religious struggle and life satisfaction. *p < .05; **p < .01

The PROCESS macro (version 3.2) (Hayes 2017) was applied to test whether self-esteem mediated the association between the four dimensions of religious struggle separately, and life satisfaction. Figure 1 exemplifies the model of relationships that depict the mediation dynamic. Religious comfort, fear/guilt, negative emotions toward God, and negative social interactions surrounding religion were expected to be the independent variables, and life satisfaction was assumed as the dependent variable. Self-esteem operated as a mediating variable.

Consequently, there were four single-level mediation models, including three-variable systems. For the present analysis, bootstrapping procedures were implemented. As proposed by Preacher and Hayes (2008), 5000 bootstrap samples and 95% confidence intervals were applied to evaluate the indirect effects that are considered significant if they do not include zero. This method is believed to be more accurate than traditional mediation analyses as it does not require the normality of the sampling distribution (Hayes 2013).

## Results

Religious comfort, fear (struggling with religious doubts), negative emotions toward God (feeling abandoned, unloved or punished by God), negative social interactions (having interpersonal conflicts related to religion), satisfaction with life, and self-esteem were tested for skewness and kurtosis to assess the normality of the variables’ distribution. We assumed values less than the ± 2 usually considered acceptable as a normal distribution (George and Mallery 2016) for skewness, and ± 3 for kurtosis (Ruppert 2004). In fact, no variables surpassed the cutoffs of ± 2 for skewness (− 0.792 to 1.749), and ± 3 for kurtosis (− 0.798 to 2.957). It is important to notice that the scores of religious comfort, fear, negative emotions toward God, and negative social interaction obtained in the present study were quite similar to the values obtained by Zarzycka (2014), Zarzycka et al. (2017), and Szcześniak et al. (2019, 2020) in different Polish samples.

Consistent with our hypotheses (H1 and H2), life satisfaction positively correlated with religious comfort (Table 1) and was negatively associated with fear/guilt, negative emotions toward God, and negative social interactions surrounding religion. These relationships were found to be statistically significant at *p* values < 0.01. Put differently, it was found that participants with higher life satisfaction were more likely to feel religious comfort, and perceive their relationship with God as close and collaborative. They were also less prompt to see God as distant or punitive, showed lower concern about being condemned by God, and experienced fewer negative feelings toward religious community members. The same pattern of results was shown in the case of self-esteem.

**Table 1:** Correlation matrix between dimensions of religious struggle, life satisfaction, and self-esteem (*N* = 607)

	1	2	3	4	5	6
1. RC						
2. FG	0.322***					
3. NEG	− 0.456***	0.193***				
4. NSIR	− 0.442***	0.083*	0.503***			
5. SWL	0.438***	− 0.200***	− 0.325***	− 0.248***		
6. SES	0.185***	− 0.360***	− 0.341***	− 0.244***	0.848***	
M	6.550	2.980	1.496	4.316	21.074	28.947
SD	3.389	2.131	1.971	2.162	6.950	6.493

*RC* religious comfort; *FG* fear/guilt; *NEG* negative emotions toward God; *NSIR* negative social interactions surrounding religion; *SWL* satisfaction with life; *SES* self-esteem; *M* mean; *SD* standard deviation

*p < 0.05; **p < 0.01; ***p < 0.001

Although there is no consensus on which VIF cutoff score is most appropriate for collinearity (Thompson et al. 2017), many authors agree that a VIF index higher than 5.0 (Hair et al. 2017) and a tolerance value of less than 0.2 imply multicollinearity (Mehmetoglu and Jakobsen 2017). Given that the analysis of multiple regression reported a VIF of 1.09–3.98 and a tolerance rate oscillating between 0.25 and 0.91, multicollinearity indexes implied little indication of multicollinearity for these data. Mahalanobis’ distance procedure was employed, using the Chi-square distribution with a very conservative probability estimate for a case being an outlier (*p* < 0.001) (Fidell and Tabachnick 2003). Only ten cases of 607 were detected as possible multivariate outliers. Nevertheless, because analyses run with and without the cases named as outliers showed that they did not have a large effect on the correlations, regressions, or mediations and did not change the results (Stevens 2009), they were not dropped from the analysis. Furthermore, Cook’s value (between 0.000 and 0.056) was well under the point at which the researcher should be concerned, that is, less than 1 (Fidell and Tabachnick 2003), indicating that the cases were not problematic in terms of having an excessive effect on the model (Lomax and Hahs-Vaughn 2012). Hierarchical regression analyses showed that neither sex, age nor the subjective assessment of one’s religious involvement made a significant unique contribution to the model, explaining only 6% of the variance (*R*^2^ = 0.057): sex (*β* = − 0.050, *t* = − 1.549, *p* = 0.122), age (*β* = − 0.007, *t* = − 0.208, *p* = 0.835), and subjective assessment of one’s religious involvement (*β* = − 0.005, *t* = − .094, *p* = 0.925). The predictors explained an additional 38% of the variance in the outcome even after checking for the effects of hypothetically confounding factors (sex, age, and subjective assessment of one’s religious involvement).

With the aim of further analysis of the relationship between the dimensions of religious strains and life satisfaction, self-esteem was implemented as a prospective mediator which could explain the process by which the independent variables (religious comfort, fear/guilt, negative emotions toward God, and negative social interactions surrounding religion) affect the dependent variable (life satisfaction). Table 2 shows the statistics related to each mediation path.

**Table 2:** The role of self-esteem in the relationship between religious struggle (religious comfort, fear/guilt, negative emotions toward God, negative social interactions surrounding religion) and life satisfaction (*N* = 607)

	a path	b path	c path	c’ path	Indirect effect and B (SE)	95% CI lower upper
1. RE–SES–LS	0.35***	0.66***	0.50***	0.26***	0.2368 (0.0575)	0.1291; 0.3564
2. FG–SES–LS	− 1.09***	0.70***	− 0.65***	0.12	− 0.7755 (0.0882)	− 0.9506; − 0.6072
3. NEG–SES–LS	− 1.12***	0.65***	− 1.14***	− 0.41***	− 0.7313 (0.1040)	− 0.9390; − 0.5345
4. NSI–SES–LS	− 0.73***	0.66***	− 0.79***	− 0.30**	− 0.4900 (0.0826)	− 0.6601; − 0.3351

1. RE–SES–LS: Religious comfort–Self-esteem–Life satisfaction; 2. FG–SES–LS: Fear/guilt–Self-esteem–Life satisfaction; 3. NEG–SES–LS: Negative emotions toward God–Self-esteem–Life satisfaction; 4. NSI–SES–LS: Negative social interactions surrounding religion–Self-esteem–Life satisfaction; a = effect of the predictor on the mediator; b = effect of the mediator on the outcome; c = effect of the predictor on the outcome; c’ = direct effect of the predictor on the outcome while controlling for the mediator

*p < .05; **p < .01; ***p < .001

The results obtained from bootstrap sampling (5000) with a 95% confidence interval indicated a significant role of self-esteem as a mediator in all of the relationships between: (1) religious comfort and life satisfaction; (2) fear/guilt and life satisfaction; (3) negative emotions toward God and life satisfaction; and (4) negative social interactions surrounding religion and life satisfaction.

There was a mediation since the indirect effect did not contain a zero, demonstrating statistically significant relationships. In fact, in all four cases, the original path c dropped to c’ as a result of including the mediator.

## Discussion

The first purpose of this study was to examine the relationship between four dimensions of religious struggles, life satisfaction, and self-esteem (H1 and H2). The second aim was to verify whether self-esteem mediated the relationships between religious struggle and satisfaction (H3). This research confirms all three hypotheses well enough and is consistent with previous studies.

In terms of the first hypothesis, all dimensions of religious struggle significantly correlated with life satisfaction. Likewise, Szcześniak et al. (2019) reported that young Polish Catholics with higher satisfaction were also more likely to declare a higher sense of trust in a loving God, and a lower sense of guilt when faced with their own transgressions. In other studies (Kent et al. 2018), religious believers who constructively adopted God’s compassion and mercy were likely to feel a greater sense of well-being. In a large and systematic overview of the literature on the psychology of religion, Koenig et al. (2001) found that life satisfaction, even after controlling for multiple covariates, significantly and positively correlated with prayer, scripture reading, religious meaning, attendance, and faith in God. However, some researchers observe that the connection between religiosity and life satisfaction is bimodal and two-dimensional (Okulicz-Kozaryn 2010). On the one hand, religious people are inclined to be either very pleased or disappointed with life, and on the other hand, religiosity has forms that help to increase or reduce life satisfaction. With respect to guilt/fear, a concern with one’s own transgressions and feelings of being unforgiven by God correlated negatively with life satisfaction. This result confirms outcomes found by Fichnam and May (2019). According to their findings, knowing that one is pardoned by God may facilitate self-forgiveness which, in turn, is positively associated with psychological well-being, and inversely linked to depressive symptoms. Moreover, awareness of divine forgiveness and self-forgiveness were significant predictors of life satisfaction. With regard to negative emotions toward God and life satisfaction, different studies showed that individuals who are disappointed or angry with God are more likely to experience reduced psychological well-being (Exline et al. 2017; Strelan et al. 2009). Concerning negative interactions with members of the religious community, Krause et al. (2000) found that unpleasant encounters with other fellows within the Church were, for adults participating in the research, a source of anxiety and distress. Conversely, people with a strong sense of belonging to a religious community declared themselves more satisfied with their lives than their non-religious counterparts (ten Kate et al. 2017). In this context, it is understandable that religious struggles in the form of guilt/fear, negative emotions toward God and religious people/institutions are associated negatively with life satisfaction and other dimensions of well-being.

As for the second hypothesis, a similar pattern of results was obtained. Self-esteem correlated significantly and positively with religious comfort, and significantly and negatively with fear/guilt, negative emotions toward God and negative social interactions surrounding religion. These results have found empirical support in a majority of studies. Koenig et al. (2001) discovered that in almost 70% of reports examined, individuals who declared themselves to be more religious, expressed higher levels of self-esteem, as well. Trevino et al. (2019) and Bryant and Astin (2008) found that religious struggle was negatively correlated with self-esteem together with other resource variables such as emotional support, optimism, and spiritual growth. Ghorbani et al. (2017) noticed that while positive religious coping was positively associated with self-esteem, negative religious coping was associated with lower self-esteem and integrative self-knowledge. These and similar results are not surprising in the context of what some authors have said about the relationship between religiosity and self-esteem. For example, according to Emmons (2001), for many people, religious principles and behaviors stand as an essential theme of their personal identity. This can be especially valid for those societies where religiosity is still an important value. In fact, some researchers (Gebauer et al. 2012; Sedikides and Gebauer 2014) reported that self-esteem among believers was higher than that of non-believers in countries which value religiosity more. Conversely, believers and non-believers did not differ in the aspect of self-esteem in nations which did not value religiosity too much. Considering that Poland is still a highly religious country (Charzyńska and Heszen-Celińska 2019), Emmon’s remark may be true for the participants of our study. Moreover, Benson and Spilka (1973) observed that for Catholic adolescents for whom religion was personally important, self-esteem correlated positively with God perceived as being caring, and negatively with God seen as a controlling, vindictive, stern, and impersonal allness. The latest research seems to confirm these results. For example, it was found that the Christian tradition, a positive image of God, and church attendance predict higher levels of self-esteem among young people (Williams et al. 2008; Robbins et al. 2007). Further, self-esteem correlated negatively with the image of God seen as controlling and impersonal. Similarly, Francis et al. (2010) claimed that the ways in which people experience themselves is associated with the way in which they suppose that God feels about them. Put differently, the authors postulate that images of God as loving and compassionate may be echoed in a more positive self-esteem, while images of God as just and demanding may be reflected in a less positive self-esteem. With respect to other aspects of religious struggle, Zinnbauer and Pargament (1998) observed that repeated or unresolved resentment toward God, which represents divine struggle, correlated with lower self-esteem. Moreover, Kent and colleagues (2018) found that individuals who felt forgiven by God and disclosed secure attachment to God had the highest levels of self-esteem. Strelan (2007) reported that proneness to guilt correlated moderately with self-esteem.

With respect to the third hypothesis, self-esteem resulted as a mediator of the relationship between religious comfort/three dimensions of religious struggle and life satisfaction. In other words, the mediating effect of overall evaluation and respect of the self implies that people with a higher sense of trust in God are more disposed to developing their self-esteem, which in turn leads to an increase of their life satisfaction. Previous studies have shown that religiosity can provide a significant basis for self-esteem (Koenig 2012). Crocker et al. (2003) suggest that self-esteem is affected not only by the approval of other people, but also by confidence that one is valuable in the eyes of a trustworthy God. A certainty of being loved and accepted by God can form in believers a foundation for a steadfast sense of self-worth (Joshanloo and Daemi 2015). People who find firm meaning in their faith are more inclined to have higher levels of self-worth (Krause 2003). This, in turn, contributes to greater life satisfaction. In fact, Krause (1992) found that self-esteem was an important intervening mechanism that may affect the relationship between religiosity and psychological well-being. At the same time, the results of the current research also imply that individuals who experience fear/guilt, negative emotions toward God or the religious community are at risk of lower self-esteem, which may lead them to have lower life satisfaction. Such findings seem to be consistent with Crocker and Wolfe’s (2001) approach, which suggests that self-esteem can be susceptible to the occurrence of different setbacks and failures in important domains of life. They are also in line with empirical outcomes which imply that God’s love is considered a relevant internal source of self-esteem (Park et al. 2004). If this love is not felt, or there is a lack of belonging to the religious community, self-esteem may diminish. Therefore, threats to the relationship with divinity, experienced through feelings of being unforgiven by, or angry with God, and adverse emotions or relations with religious “others” may reduce self-esteem, contribute to a sense of powerlessness (Exline and Rose 2013), and result in lower life satisfaction.

## Conclusion

The study enlarges our understanding of religious struggles and self-esteem among Polish Christians and confirms prior research about the relationship between religious stains and life satisfaction. Though the correlational and mediatory character of our results does not allow us to draw causal deductions, the outcomes denote that the co-occurrence of self-esteem in the context of religious struggles may be important for life satisfaction, consolidating the connection between religious comfort and life satisfaction, and alleviating the link between fear, negative emotions toward God or the religious community, and life satisfaction.

However, this research has a number of limitations. Firstly, given that the participants were recruited mainly via online Internet communities and groups for Christians, it cannot be expected that they reflect the general Christian population. Specifically, the respondents representing late adulthood are less likely to use Internet resources. It would be enriching to extend the sample to a greater group of middle and late adults, as older people are often confronted with challenges that are sources of religious doubts (Thauvoye et al. 2019). Secondly, since the data were collected through self-reports, the results could be influenced by social desirability bias. Hence, we suggest that in subsequent studies, researchers might apply questionnaires that aim to monitor the respondents’ inclination to present themselves in the most favorable manner. Thirdly, although we found support for the mediating role of self-esteem on the relationship between religious struggles and life satisfaction, we did not measure causation in these associations because of the cross-sectional design assumed in the current study. Nevertheless, we tested mediation in the cross-sectional data presenting a fitting rationale for the posited mediation process. In the future, it would be important to use a longitudinal design to better grasp how religious struggles might impact life satisfaction through self-esteem.
